# Prediction of Translational Regulation by Network Interaction in Synaptic Plasticity Induced with *Centella asiatica*

**DOI:** 10.1155/2023/4199614

**Published:** 2023-06-24

**Authors:** Nurhadi Ibrahim, Ibrahim Nadian, Dimas R. Noor, Fadilah Fadilah

**Affiliations:** ^1^Department of Medical Physiology and Biophysics, Faculty of Medicine, Universitas Indonesia, Jakarta 10430, Indonesia; ^2^Department of Medical Chemistry, Faculty of Medicine, Universitas Indonesia, Jakarta 10430, Indonesia; ^3^Human Cancer Research Center, Indonesian Medical Education and Research Institute, Faculty of Medicine, Universitas Indonesia, Jakarta, Indonesia; ^4^Bioinformatics Core Facilities-IMERI, Faculty of Medicine, Universitas Indonesia, Jakarta 10430, Indonesia

## Abstract

**Background:**

Recently, human life expectancy, aging, and age-related health disorders, especially neurodegenerative diseases such as Alzheimer's disease (AD), have increased. The increasing number of AD patients causes a heavy social and economic burden on society. Since there is no treatment for AD, utilization of natural products is currently accepted as an alternative or integrative treatment agent against AD.

**Methods:**

Selection of protein databases related to synaptic plasticity was obtained from a gene bank. The protein-protein interaction (PPI) analysis was performed using Cytoscape 3.9.1. Prediction of *Centella asiatica* target constituents and their relationship with target synaptic plasticity was performed using STITCH, followed by GO and KEGG pathway enrichment analysis and molecular binding of ligands to presynaptic and postsynaptic receptors afterwards.

**Results:**

From the protein database, 446 protein coding genes related to synaptic plasticity were found. PPI and KEGG pathway analysis showed potentiality to inhibit AKT and mTORC1 pathways. The targeted proteins were TSC1, Rheb, and FMRP.

**Conclusion:**

This study showed potentiality of *Centella asiatica* in AD through its binding to several proteins such as TSC1, Rheb, and FMRP. This compound in *Centella asiatica* was able to bind to the AKT1 and mTOR signaling pathways. *Centella asiatica* may behold greater potency in AD therapy.

## 1. Introduction

*Centella asiatica* (CA), also known as Asian pennywort or Gotu kola, is a plant with a rich history of use in Ayurvedic and traditional Chinese medicine. It contains several biologically active compounds, including alkaloids, flavonoids, phenols, tannins, and terpenoids. The triterpene saponins found in CA, such as asiatic acid, asiaticoside, madecassic acid, and madecassoside, are particularly noteworthy for their therapeutic potential. These compounds possess various biological activities, including anticancer, wound-healing, antibacterial, antidiabetic, anti-inflammatory, and antioxidant properties. Additionally, CA has been valued for its cognitive-enhancing effects [1].

The prevalence of neurodegenerative diseases, including Alzheimer's disease (AD), is increasing due to human life expectancy and aging [2]. Individuals with AD experience symptoms such as memory and cognitive impairments, sleep disturbances, and activation of microglia, a type of brain cell [3]. The onset of sporadic AD, the most common form, is believed to be influenced by genetic and environmental factors [4]. AD is associated with oxidative stress, neuroinflammation, and mitochondrial dysfunction [4, 5]. However, the mechanisms underlying AD pathogenesis are not fully understood, highlighting the need to target multiple mechanisms for prevention and treatment [6].

Natural products have a long history of use in medicine, and their potential as neuroprotective agents in AD has gained significant attention [7]. CA and its bioactive compounds have been investigated for their neuroprotective effects in AD pathology [8, 9]. Previous studies have shown the potency of CA in reducing neuroinflammation and promoting neuronal cell survival [8]. In silico studies have also demonstrated potential molecular interactions between CA compounds and AD proteins [9].

Considering the limited effectiveness of current AD treatment options, there is a growing interest in exploring alternative strategies [7]. Our study aims to enhance our understanding of the therapeutic potential of *Centella asiatica* in AD by employing various bioinformatics approaches, as described previously [10]. Through compound identification, analysis of biological markers, and molecular docking, we aimed at investigating the effectiveness of *Centella asiatica* in inducing synaptic plasticity, a crucial mechanism for cognitive function. By exploring these mechanisms, we hope to contribute to the development of alternative or complementary treatments for AD and other neurodegenerative disorders.

## 2. Methods

### 2.1. Selection of Protein Databases Related to Synaptic Plasticity

The protein database was retrieved from GenBank using several keywords related to synaptic plasticity. These methods employed a standard bioinformatics approach to reveal the biological network that has been previously described [10]. These keywords were taken from the MeSH database (https://www.ncbi.nlm.nih.gov/mesh/?term=neuroplasticity). The data were downloaded in a comma-separated value format (484 genes) and processed in Excel to delete noncoding protein data, resulting in a total of 466 protein-coding genes. Then, the data were submitted to Cytoscape 3.9.1 software (https://cytoscape.org/).

### 2.2. Protein-Protein Interaction (PPI) Analysis and Prediction of *Centella asiatica* Target Constituents and Their Relationship with Target Synaptic Plasticity

Protein-protein interaction (PPI) was obtained using the STRING application found in Cytoscape. All proteins were submitted to the STITCH protein query [11]. Specimens were set as *Homo sapiens* and filtered for PPI with interaction values ≥0.900. From the obtained proteins, a query was performed using a STITCH compound query containing proteins and molecules from *Centella asiatica*. A protein-compound interaction filter and enrichment were then performed using the STITCH database (https://stitch-db.org/), selecting several proteins, with *Homo sapiens* as the selected species.

### 2.3. GO and KEGG Pathway Enrichment Analysis

Gene Ontology (GO) and Kyoto Encyclopedia of Genes and Genomes (KEGG) pathway analyses were performed using Enrichr (https://amp.pharm.mssm.edu/Enrichr/) for clusters obtained from gene expression results [12]. Enrichr is a web-based tool that allows the evaluation of annotations with an extensive set of different gene libraries. Process analysis was conducted on each of the GO and KEGG pathways. The significant pathways were selected with an adjusted *p* value threshold of <0.05. The most significant biological GO processes (adjusted *p* value <0.05) and KEGG pathways in each group of the 2021 dataset were identified.

### 2.4. Molecular Binding of Ligands to Presynaptic and Postsynaptic Receptors

Molecular docking of ligands to presynaptic and postsynaptic receptors from PDB TSC (5EJC), Rheb (6BTO), and FMRP (4QVZ) was performed. The docking processes were conducted using AutoDock 6, including the coordination stage with a receptor grid generation made in the program. The van der Waals scaling factor was changed to 1, and the partial charge cutoff was set to 0.25. All ligands were molecularly anchored to the target using an application algorithm. Glide dock settings allowed a maximum of 5000 poses per ligand, which were then filtered to 1000 poses per ligand for energy minimization. Finally, 100 poses per ligand were selected based on the highest ranking of the E-model values. The molecular docking process has been described in a previous report [10].

### 2.5. Statistical Analysis

Statistical methods were employed to analyze the data and assess the significance of the results. Descriptive statistics were used to summarize the protein-coding genes and their associations with synaptic plasticity. Enrichment analyses identified significant pathways based on adjusted *p* values. The molecular docking process involved statistical selection of poses based on the highest ranking of the E-model values. These statistical approaches provided valuable insights into the relationships and potential significance of the findings in this study.

## 3. Results and Discussion

### 3.1. Asiaticoside and Betulinic Acid Are the 2 Constituent Molecules of *Centella asiatica* That Can Interact with Synaptic Plasticity

In this study, we identified 336 target proteins associated with synaptic plasticity that have a strong relationship with each other. These interactions were mapped through the STRING protein query in Cytoscape 3.9.1 software. This study showed that, out of the 336 proteins, we identified the relationship with 9 constituent molecules of *Centella asiatica*. From these, 5 proteins were able to bind at least 2 constituent molecules of *Centella asiatica*. These proteins were BDNF, CCL2, and VEGFA, which bind to asiaticoside. Betulinic acid can also bind to AKT1 and MAPK1. From the STITCH enrichment results, there were 15 proteins that were related to each other, as well as 1 protein-component interaction, namely, betulinic acid and mTOR.

**Figure 1:**
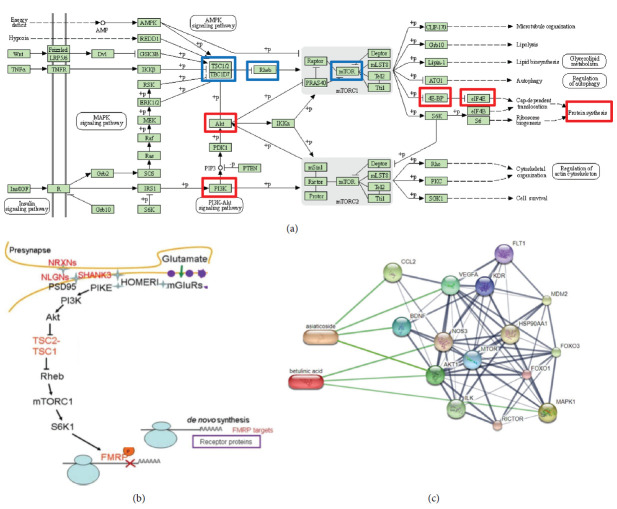
Pathway of synaptic plasticity. (a) The red boxes represent targeted proteins (TSC1 and Rheb), while the blue boxes represent the targeted pathways. (b) A modified version from Khlebodarova et al. and (c) adapted from [13]. The figure demonstrates the interaction of asiaticoside and betulinic acid with proteins involved in synaptic plasticity. Specifically, BDNF, CCL2, and VEGFA bind to asiaticoside, while betulinic acid can also bind to AKT1 and MAPK1.

From KEGG enrichment, it was found that 10 out of 15 proteins had important contributions to the PI3K-AKT signaling pathway and that 5 out of 15 proteins contributed to the mTOR pathway. From the enrichment molecular function, it was found that all proteins act as binding pathways for other proteins and that 10 out of 15 proteins play a role in binding anions and carbohydrates. Based on the biological enrichment process, we found proteins that play a role in regulating cell death (AKT1, BDNF, CCL2, FOXO3, ILK, and VEGFA) and 4 proteins important for the projection of differentiation and morphogenesis of nerve cells (HSP90AA1, ILK, MAPK1, and VEGFA). In Figure 1(a), we show several proteins and signaling that are potentially targeted.

### 3.2. Molecular Docking Results Show the Role of Compounds in *Centella asiatica* with TSC1, Rheb, and FMRP

**Figure 2:**
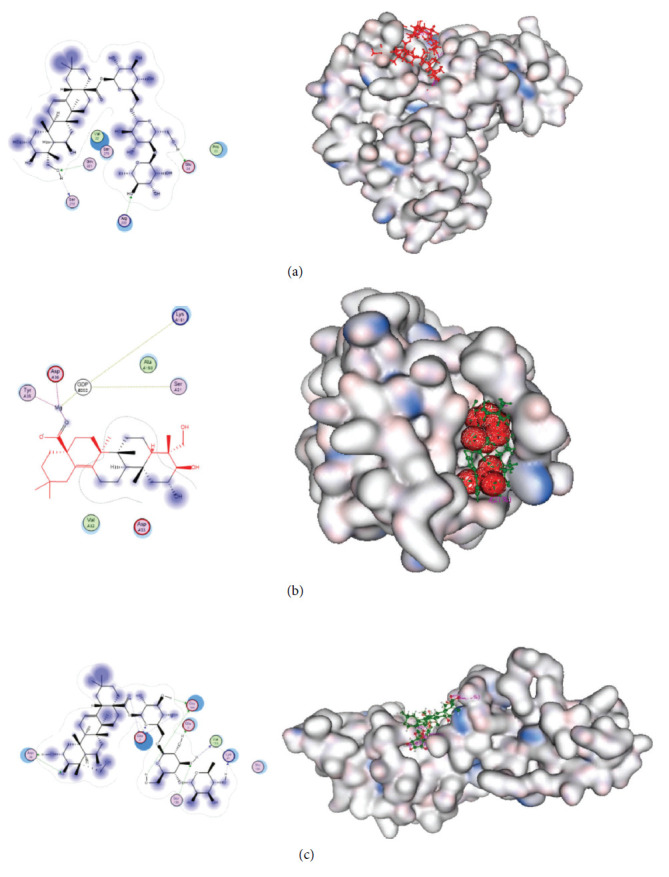
Results of molecular docking on AKT signaling-related proteins. Complex of TSC1-asiaticoside B (a), complex of Rheb-Centella sapogenol (b), and interaction of asiaticoside B compounds at the N terminal of FMRP (c).

The molecular docking results reveal the interactions of asiaticoside compounds, including asiaticoside B, with various proteins, such as TSC1, Rheb, and FMRP, in the AKT pathway. Specifically, asiaticoside B interacts with TSC1 at residues Glu24, Ser218, Gln221, and Arg222, as well as with Centella sapogenol via residues Tyr35, Asp36, and Lys151. Moreover, asiaticoside B interacts with FMRP at residues Glu2, Glu3, Lys21, Asn34, Glu144, Ser158, Val159, and Ala139, while asiaticoside interacts at residues Glu2, Glu3, Lys21, Asn34, Glu144, Ser158, Val159, and Ala139. These interactions are presented in Table 1, and the docking results on a 3-dimensional structure are shown in Figures 2(a), 2(b), and 2(d). In addition, Figure 2(c) illustrates the interaction of asiaticoside B compounds at the N-terminal of FMRP.

#### 3.2.1. Complex of TSC-Asiaticoside

The analysis of inhibitor compounds from the extract of CA revealed that asiaticoside B exhibited the lowest free energy (∆G) of −5.3297 kcal/mol when binding to the TSC1 receptor. Asiaticoside B demonstrated superior affinity compared to other compounds. Visualizing the results, it was observed that asiaticoside B formed hydrogen bonds and hydrophobic interactions with active sites consisting of Glu24, Ser218, Gln221, and Arg222 amino acid residues.

TSC serves as a negative regulator of Rheb and mTORC1, contributing to cellular stress-related pathways as depicted in Figure 1. Fitzian et al. demonstrated the role of TSC1 in regulating TSC complexes and mTORC1 activity through lysosomal phosphatidylinositol. AKT/mTOR activation has been previously associated with Alzheimer's disease (AD), with downstream targets such as 4EBP1 showing increased activity [14]. Dysfunctions in the AKT/mTOR pathway have been identified as risk factors for AD [15]. A previous study by Pang et al. reported the involvement of PI3K/AKT/mTOR in maintaining a balance between autophagy and apoptosis. Inhibition of AKT-mTOR signaling has been shown to increase autophagy and decrease apoptosis processes [15]. Liu et al. reported that inhibiting cellular apoptosis and increasing autophagy can improve mitochondrial activity and restore motoric function in Parkinson's disease [16].

mTOR is activated by several signaling molecules, including PI3K/AKT, which leads to the cascade of 4EBP1 and p70S6, resulting in tau hyperphosphorylation and the formation of amyloid beta plaques [17, 18]. In addition, the accumulation of amyloid beta can impact mTOR. Previous studies have highlighted the contribution of mTOR to tau pathology and the potential of targeting this pathway to reduce tau pathology [18]. mTOR signaling has been implicated in AD, displaying a dual role in neurodegenerative disease. It has been found to increase during neurofibrillary degeneration characterized by tau phosphorylation, while an opposing effect has also been described [19].

#### 3.2.2. Complex of Rheb-Centella Sapogenol

The analysis of inhibitor compounds from the extract of CA revealed that asiaticoside B exhibited the lowest free energy (∆G) of −5.8297 kcal/mol when binding to the RheB receptor. Asiaticoside B compounds have a known higher affinity compared to other compounds. Visualization results indicated that amino acid residues interacted through hydrogen bonds and hydrophobic interactions with active sites consisting of Glu24, Ser218, Gln221, and Arg222.

RheB is a GTP-binding protein that is abundantly expressed in humans and mammals. This protein has been found to regulate the mTOR pathway [20]. Recent reports by Shams et al. demonstrated RheB as a novel candidate for inhibiting mTORC1 [17]. These studies also revealed that RheB can directly bind to mTORC1 [17]. The ability of RheB to regulate mTORC1 complexes is associated with fibrotic and degenerative diseases, including cancers and neurodegenerative conditions. Mahoney et al. reported the potential therapeutic utility of RheB signaling in inhibiting mTORC1 signaling [18]. Therefore, the molecular interactions between Centella sapogenol and RheB increase the possibility of CA in AD therapy.

#### 3.2.3. FMRP-Asiaticoside

The analysis of inhibitor compounds from the extract of CA revealed that asiaticoside B exhibited the lowest free energy (∆G) of −14.3521 kcal/mol and a pKi of 11.258 when binding to the FMRP receptor (Table 1). Asiaticoside B compounds are known to have a better affinity compared to other compounds. Visualization results indicated that amino acid residues interacted through hydrogen bonds and hydrophobic interactions with active sites consisting of Glu2, Glu3, Lys21, Asn34, Glu144, Ser158, Val159, and Ala139.

Fragile *X* mental retardation protein (FMRP) has been previously reported to be related to autism and intellectual abilities. This protein selectively binds RNA through its amino terminal-C and arginine-glycine-glycine (RGG) terminal, with the amino terminal having the ability to bind RNA without functional motives. Recently, it has been shown to affect DNA damage in prasynaps and modulate the duration of potential actions [21]. FMRP also plays a role in regulating specific mRNA expression around synapses in response to external stimuli. The absence of this protein causes abnormal translational expression, leading to alterations in synaptic plasticity [19]. Inhibition of FMRP binding to BC-1 noncoding RNA was shown to suppress APP translation and restore amyloid beta levels. Bleuzé et al. demonstrated this improvement in spatial learning and memory impairments in AD mouse models [19, 22].

### 3.3. Implications for Alzheimer's Disease Prevention

The findings of this study have important implications for the prevention of Alzheimer's disease. The compounds asiaticoside and betulinic acid found in CA demonstrate promising potential in combating this neurodegenerative disorder. Asiaticoside's interactions with BDNF, CCL2, and VEGFA indicate its ability to support neuronal growth, reduce neuroinflammation, and improve vascular health in the brain. Similarly, Betulinic acid's interactions with AKT1 and MAPK1 suggested its involvement in promoting neuronal survival, synaptic plasticity, and cognitive functions.

These results shed light on the underlying mechanisms through which *Centella asiatica*'s compounds may exert their protective effects. The molecular docking results provide further support for the therapeutic value of asiaticoside B, which demonstrates interactions with proteins associated with cellular stress, mTOR signaling, and synaptic plasticity. These findings underscore the potential of *Centella asiatica*'s compounds in preventing Alzheimer's disease by targeting key proteins and pathways involved in synaptic plasticity. Further investigations are warranted to validate and translate these findings into potential therapeutic strategies for Alzheimer's disease.

## 4. Conclusion

Our study utilized a range of bioinformatics approaches to explore the potential of *Centella asiatica* in Alzheimer's disease (AD) management. We identified specific compounds in *Centella asiatica* that exhibit binding interactions with crucial proteins involved in AD pathways, namely, TSC1, Rheb, and FMRP. These interactions suggest the potential of *Centella asiatica* to modulate signaling pathways, enhance autophagy, and regulate amyloid beta levels. The findings from this study provide valuable insights for further research and development of targeted therapeutics, offering new possibilities for effective AD management and potentially slowing down disease progression.

## Figures and Tables

**Table 1:** Molecular docking of compounds in *Centella asiatica* with proteins to several proteins in AKT signaling.

No.	Target	Compounds	*S* (kkal/mol)	pKi	Hydrogen interaction
	AKT				
1	TSC1	Asiatic acid	−3.4194		Arg375, Lys594
		**Asiaticoside B**	**−5.3297**		**Glu24, Ser218, Gln221, Arg222**
		Asiaticoside	−2.8291		Lys394
		Betulinic acid	−2.4530		Asn174
		Centella sapogenol	−3.5761	7.472	Asn174
		Isothankunic acid	−2.3297		Lys594
		Madasiatic acid	−2.2388	8.903	Asn374, Gln221
		Madecassic acid	−3.0867		Arg375

2	Rheb	Asiatic acid	−4.1194	4.561	Ser130, Arg126
		**Asiaticoside B**	**−5.8297**	**2.391**	**Asp36, Lys151**
		Asiaticoside	−3.291		Lys355
		Betulinic acid	−5.5530		
		**Centella sapogenol**	**−6.5061**	**4.391**	**Tyr35, Asp36, Lys151**
		Isothankunic acid	−4.2197	4.391	Lys169
		Madasiatic acid	−3.1288		Lys319
		Madecassic acid	−3.0867		Lys151

3	FMRP	Asiatic acid	−9.8436		His528
		**Asiaticoside B**	**−14.3521**	**11.258**	**Glu2, Glu3, Lys21, Asn34, Glu144, Ser158, Val159, Ala139**
		**Asiaticoside**	**−10.5325**		**Thr22, Glu144, Ser158, Val159**
		Betulinic acid	−10.8116	**6.321**	Arg681, Arg682
		Centella sapogenol	−7.6863		Arg682
		Isothankunic acid	−8.6692		—
		Madasiatic acid	−9.9219		Arg681, Arg682
		Madecassic acid	−7.1651		—

Bold or mark important parameters as well as amino acid residues.

## Data Availability

The data supporting the findings of this study are available upon request from the corresponding author.
